# Identification and Diagnosis of Cerebral Stroke through Deep Convolutional Neural Network-Based Multimodal MRI Images

**DOI:** 10.1155/2021/7598613

**Published:** 2021-07-20

**Authors:** Yanyan Pan, Huiping Zhang, Jinsuo Yang, Jing Guo, Zhiguo Yang, Jianbing Wang, Ge Song

**Affiliations:** ^1^Department of Neurology, Baoji High Tech Hospital, Baoji 721000, Shaanxi, China; ^2^Department of Neurosurgery, People's Hospital of Hanzhong City, Hanzhong 723000, Shaanxi, China

## Abstract

This study aimed to explore the application value of multimodal magnetic resonance imaging (MRI) images based on the deep convolutional neural network (Conv.Net) in the diagnosis of strokes. Specifically, four automatic segmentation algorithms were proposed to segment multimodal MRI images of stroke patients. The segmentation effects were evaluated factoring into DICE, accuracy, sensitivity, and segmentation distance coefficient. It was found that although two-dimensional (2D) full convolutional neural network-based segmentation algorithm can locate and segment the lesion, its accuracy was low; the three-dimensional one exhibited higher accuracy, with various objective indicators improved, and the segmentation accuracy of the training set and the test set was 0.93 and 0.79, respectively, meeting the needs of automatic diagnosis. The asymmetric 3D residual U-Net network had good convergence and high segmentation accuracy, and the 3D deep residual network proposed on its basis had good segmentation coefficients, which can not only ensure segmentation accuracy but also avoid network degradation problems. In conclusion, the Conv.Net model can accurately segment the foci of patients with ischemic stroke and is suggested in clinic.

## 1. Introduction

Globally, precision treatment of brain diseases has become a research hotspot. Under normal circumstances, brain diseases generally fall into two categories, namely, acute brain diseases and chronic brain diseases [1]. The so-called acute brain disease refers to cerebrovascular diseases of a sudden onset, and stroke is a most typical one. Clinical data show that stroke has become the first cause of deaths in China, and the number of patients dying of stroke in China is 3 million each year [2]. It is reported that the mortality rate of cardiovascular diseases in China is 271/100,000 and the mortality rate of stroke is 140/100,000. Stroke can be divided into two types: ischemic and hemorrhagic strokes, and ischemic stroke accounts for 87% [3].

Ischemic stroke refers to the gradual necrosis of brain cells arising from the lack of oxygen in the brain tissue due to insufficient blood supply, thereby forming an irreversible infarct core. If blood flow is restored in time, irreversible brain damage may be avoided. This potentially reversible tissue area surrounding the core of ischemic damage is called the “ischemic penumbra.” According to the onset time, an onset within 0–24 h is called the acute stroke, an onset within 24 h-2 w is called the subacute stroke, and an onset during a period over 2 w is called the chronic stroke. Patients with an acute stroke are manifested as focal neurological impairment, such as aphasia, hemianopia, and loss of sensation. These symptoms usually develop into chronic diseases, such as dementia and hemiplegia, seriously affecting the quality of life and causing huge economic burdens on patients [3]. Hence, it is a must to diagnose and treat acute stroke in a timely manner. Generally, it requires doctors to make an accurate diagnosis of stroke within 3 hours. Computed tomography (CT) and magnetic resonance images (MRI) are commonly used to observe and analyze the lesions of stroke patients. However, as sample size expands, the workload of doctors to read films is increasing, and the results of manual reading are always influenced by subjective factors such as the doctor's experience. Therefore, it is very necessary to develop an automatic diagnosis algorithm to assist doctors in diagnosing and analyzing the lesions of stroke patients. Compared with traditional diagnosis methods, automatic segmentation algorithms are characterized by high accuracy, high efficiency, objective analysis of lesions, strong repeatability, and scalability. Because of the complexity of brain diseases, clinicians often adopt multimodal MRI that can comprehensively reflect disease information [4], which was the focus of the study.

Recently, artificial intelligence and big data have found broad applications in the medical field, but how to extract effective features from massive medical images is the focus of current research. Deep learning is an emerging medical image data processing method. Traditional learning algorithms have weak feature expression ability due to the shallow structure of the training model, while deep learning makes up for the shortcomings of traditional learning algorithms because it is equipped with an advanced central processing unit, an improved machine algorithm, and massive medical images. It transforms the manual feature extraction into automatic machine extraction, reducing manual labor and heightening the accuracy of feature extraction. Based on the abovementioned advantages, it has received extensive attention. According to whether the training dataset is labeled or not, machine learning algorithms can be divided into two categories: supervised learning and unsupervised learning. Research has found that the supervised deep learning algorithm performs better in image analysis. In particular, the deep convolutional neural network (Conv.Net) is the most valuable and potential method for image processing and analysis [5]. Conv.Net is an improved neural network proposed in 1989 and became popular in 2012. It mainly consists of the input layer, convolutional layer, downsampling layer, fully connected layer, and output layer. Gradually, a variety of deep CNN models have been established. Statistics have shown that their feature recognition error rate has been reduced to 3.5%. As mentioned above, the Conv.Nets have demonstrated great capabilities in image processing and feature extraction [6]. They are extensively used in image segmentation, image classification, image registration, and target detection. In the research of stroke disease, the existing Conv.Nets focus on the two-dimensional slice information and ignore the three-dimensional information. Although they have achieved good results, they are not comprehensive, in-depth, and accurate enough [7–9]. In the study, the multimodal magnetic resonance imaging (MRI) images were segmented by the Conv.Net, and the segmentation effects were compared with other algorithms, so as to find an optimal algorithm to assist doctors in diagnosing strokes.

## 2. Materials and Methods

### 2.1. Research Subjects and Grouping

Patients with acute stroke in hospital from January to April 2020 were selected as the research subjects. Each patient underwent seven MRI examinations in the acute phase, including T1c, T2, CBF, CBV, DWI, Tmax, and TTP. The registration and skull dissection were performed in each MRI mode, and the image resolution was 2 × 2 × 2 mm^3^. The MRI data of 30 patients were used as the training set, and 30 patients undergoing 7 MRI examinations were in the test set. The “penumbra” was marked manually by an expert based on the perfusion and diffusion images, as per the currently recognized linear threshold.

### 2.2. CNN Algorithm

**Figure 1:**
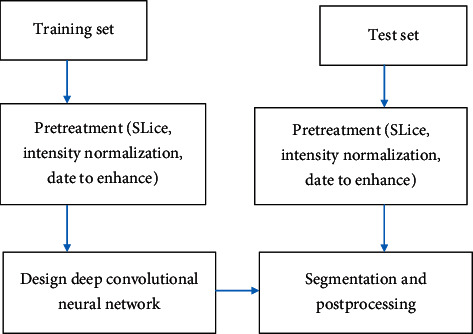
The flow chart of the segmentation algorithm based on the cascaded 3D deep residual network.

Figure 1 is the flow chart of the segmentation algorithm based on the cascaded 3D deep residual network. First, the 3DMRI images of the training set and the validation set are preprocessed. Then, the preprocessed brain MRI slice images are input into the 2D full convolutional neural network for training. Finally, the test set MRI slice images are segmented. In the study, the TTP mode is selected. During the training process, if all brain cross-sectional layers are input, negative samples will interfere with the network. Therefore, only the layers with lesions are selected as the training samples.

### 2.3. Image Data Preprocessing

**Figure 2:**
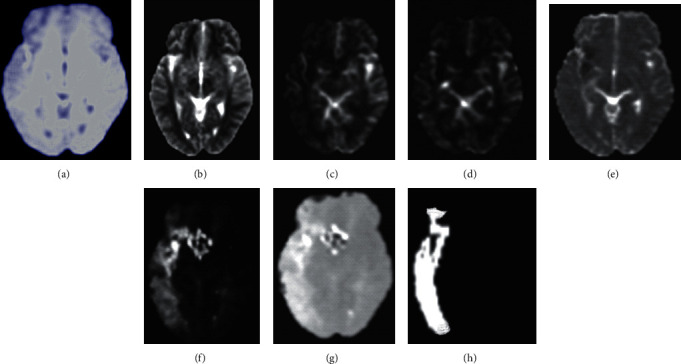
Cross sections and labels under 7 MRI modalities. (a) T1C; (b) T2; (c) CBF; (d) CBV; (e) DWI; (f) TMax; (g) TTP; and (h) GT.

The specific process of image data preprocessing is as follows. Slicing: cross-sectional slices of 50 TTP modal 3DMRI images in the training set and the test set are required, as shown in Figure 2. Intensity normalization: through the image conversion method, the gray difference with diagnostic value is retained, and the gray inconsistency is reduced and eliminated. Specifically, the average gray value of the image is subtracted and the result is then divided by the variance, so that the gray distribution reaches a normal distribution with a mean value of 0 and a standard deviation of 1. Data augmentation: more training data are added to the original data to improve network performance and avoid overfitting. Specifically, all data are rotated by ±40° to enlarge the training data by 3 times.

### 2.4. Cascaded 3D Deep Residual Network

The Conv.Net algorithm mainly consists of the convolutional layer, pooling layer, fully connected layer, and Softmax classification layer. The calculation method of the convolution process is as follows:

(1) $$\begin{array}{c} x_{j}^{i}=f\left(\underset{i\in M_{j}}{\sum }x_{j}^{l-1}•K_{ij}^{l}+b_{j}^{l}\right), \end{array}$$

where *l* is the number of layers, *K* is the convolution kernel, *x*_*j*_^*l*−1^ is the feature map output by the previous layer, *K*_*ij*_^*l*^ is the convolution kernel weight, *b* is the bias value, and *f*(•) is the activation function. The convolution operation has three modes: full convolution, same convolution, and valid convolution.

(2) y=convx,w,′full'=y1,…yt,…,yn+m−1∈R,yt=∑i=1mxt−i+1•wi, t=1,2,…,n+m−1,y=convx,w,‘same'=centerconvx,w,‘full',n∈R,y=convx,w,′valid'=yl,…yt,…,yn+m−1∈R,yt=∑i=1mxt+i−1wi, t=1,2,…,n+m−1.

**Figure 3:**
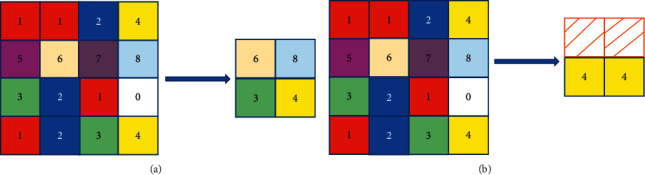
Schematic diagram of pooling. (a) Maximum pooling; (b) mean pooling.

The pooling layer can reduce the possibility of overfitting and improve the fault tolerance of the model, expressed as follows:

(3) $$\begin{array}{c} x_{j}^{l}=f\left(\beta_{j}^{l}do\;wn\left(x_{j}^{l-1}\right)+b_{j}^{l}\right), \end{array}$$

where *do*  *wn*(•) is the downsampling function and *β* and *b* are the multiplicative bias and the additive bias, respectively. There are two common pooling operations in convolutional neural networks, namely, mean pooling and max pooling. Mean pooling is to output the mean value in the filter range as the pooling output. Maximum pooling is to use the maximum value in the filter range as the pooling output, as shown in Figure 3.

Fully connected process: in a convolutional neural network, the output result of the previous layer is encoded into a one-dimensional vector. The fully connected layer is defined as follows:

(4) $$\begin{array}{c} x^{l}=f\left(w^{l}x^{l-1}+b^{l}\right), \end{array}$$

where *w*^*l*^ is the network weight coefficient, *x*^*l*−1^ is the output feature map of the upper layer, and *b*^*l*^ is the bias item of the fully connected layer.

Softmax classification layer: it is a multiclassifier which can complete more than 2 types of classification tasks and convert multiple outputs into probability values in the (0, 1) interval. In logistic regression, the training set is *T*={(*x*^(1)^, *y*^(1)^),…, (*x*^(*m*)^, *y*^(*m*)^)}, the input sample is *x*^*i*^ ∈ *R*^*n*^, and *y*^(*i*)^ is the sample label, *y*^(*i*)^ ∈ {0,1}. The hypothesis function is defined as follows:

(5) $$\begin{array}{c} h_{\theta }\left(x\right)=\frac{1}{\left(1+e^{-\theta_{X}^{T}}\right)}. \end{array}$$

The cost function value *J*(*θ*) is minimized.

(6) $$\begin{array}{c} J\left(\theta \right)=-\frac{1}{m}\left[\sum_{i=1}^{m}y^{\left(i\right)}\log\;h_{\theta }\left(x^{\left(i\right)}\right)+\left(1-y^{\left(i\right)}\right)\log\left(1-h_{\theta }\left(x^{\left(i\right)}\right)\right)\right]. \end{array}$$

The calculation equation of Softmax is as follows:

(7) $$\begin{array}{c} h_{\left(\theta \right)}\left(x^{\left(i\right)}\right)=\left[\begin{array}{c} p\left(y^{\left(i\right)}=1|x^{\left(i\right)},\theta \right)\\ p\left(y^{\left(i\right)}=2|x^{\left(i\right)},\theta \right)\\ \cdots \\ p\left(y^{\left(i\right)}=k|x^{\left(i\right)},\theta \right) \end{array}\right]=\frac{1}{\sum_{j=1}^{k}e^{\theta_{j}^{T}x^{\left(i\right)}}}\left[\begin{array}{c} e^{\theta_{1}^{T}x^{\left(i\right)}}\\ e^{\theta_{2}^{T}X^{\left(i\right)}}\\ \cdots \\ e^{\theta_{K}^{T}x^{\left(i\right)}} \end{array}\right]. \end{array}$$

It learns on the training sample *T*, to minimize the damage function of Softmax. The minimum loss function is expressed as follows:

(8) $$\begin{array}{c} J\left(\theta \right)=-\frac{1}{m}=\left[\sum_{i=1}^{m}\sum_{j=1}^{k}1\left\{y^{\left(i\right)}=j\right\}\log\frac{e^{\theta_{i}^{T}x^{\left(i\right)}}}{\sum_{i=1}^{k}e^{\theta_{i}^{T}x^{\left(i\right)}}}\right], \end{array}$$

where 1{*y*^(*i*)^=*j*} indicates that if *y*=*j*, the value is 1; otherwise, it is 0. A smaller loss function indicates that it is closer to the expected target.

Additionally, the level set, FCM, CNN, 3D-cascade-U-Net, and asymmetric 3D-cascade-U-Net are used to segment multimodal MRI images of stroke patients, which are not discussed in detail due to the word limitation.

### 2.5. Evaluation Indexes of Segmentation Results

The performance of this algorithm is evaluated factoring in DICE coefficient, precision, sensitivity, average symmetric surface distance (ASSD), and Hoffman distance. The area correctly segmented as a lesion is defined as true positive (TP); the area that is incorrectly segmented as a lesion is a false positive (FP); and the lesion area that is incorrectly segmented as a nonlesion is defined as a false negative (FN). The DICE coefficient, accuracy, and sensitivity are expressed as follows:

(9) $$\begin{array}{c} \text{DICE}=\frac{2\text{TP}}{\text{TP}+\text{FP}+\text{FN}}\text{,}\\ \text{precision}=\frac{\text{TP}}{\text{TP}+\text{FP}}\text{,}\\ \text{recall}=\frac{\text{TP}}{\text{TP}+\text{FN}}\text{.} \end{array}$$

They are used to evaluate the accuracy of the segmentation results through volume overlap. However, because of boundary differences resulting from overlap, the ASSD and the Hoffman distance are used to measure this difference. The smaller values of these two indicators suggest higher accuracy. Let A be a point on the surface of the lesion label and B be a point on the surface of the segmentation result. Then, the ASSD is defined as follows:

(10) $$\begin{array}{c} \text{ASD}\left(A,B\right)\frac{\sum a_{\in }A\min_{b\in B}d\left(a,b\right)}{\left|A\right|},\\ \text{ASSD}\left(A,B\right)=\frac{\text{ASD}\left(A,B\right)+\text{ASD}\left(B,A\right)}{2}. \end{array}$$

The Hoffman distance is expressed as follows:

(11) $$\begin{array}{c} \text{HD}\left(A,B\right)=\max\left\{\underset{a\in A}{\max}\underset{b\in B}{\min}d\left(a,b\right)\underset{a\in A}{\max}\underset{b\in B}{\min}d\left(a,b\right)\right\}. \end{array}$$

## 3. Results

### 3.1. Multimodal MRI Examination Results

**Figure 4:**
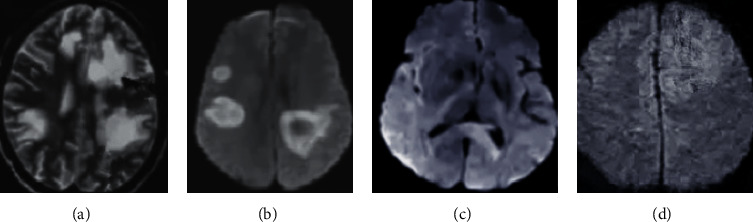
Partial MRI results of stroke patients. (a) TI; (b) T2; (c) T1C; and (d) flair.

Figure 4 shows partial MRI results of stroke patients. It was inferred that the T1 mode played an important role in reflecting the brain structure, the T2 mode can clearly reflect the lesion area, the T1C mode can enhance the T1 mode, and the flair mode can reflect disease information to the greatest extent. All in all, multimodal MRI can accurately, clearly, and comprehensively reflect disease information from multiple dimensions, providing sufficient data for disease diagnosis and analysis.

### 3.2. Segmentation Results of the Cascaded 3D Deep Residual Network Algorithm

**Figure 5:**
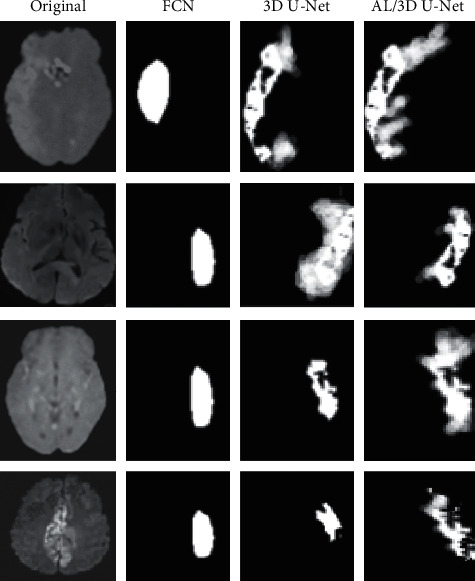
Segmentation results of the test set.

**Figure 6:**
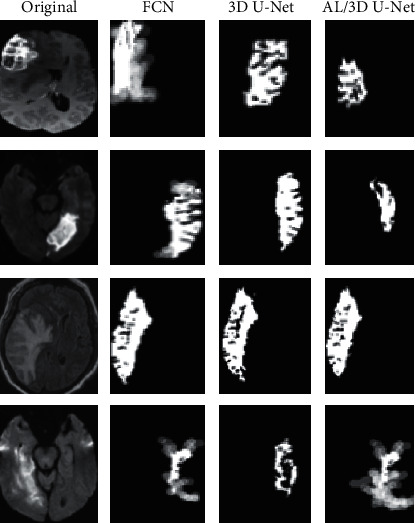
Segmentation results of the training set.

Figures 5 and 6 show the segmentation results of test set and training set. It was noted that the segmentation results by the cascaded 3D deep residual network algorithm were highly consistent with the lesion labels drawn by the expert. Hence, it can not only accurately locate the foci but also clearly reflect its edge and texture characteristics, which was superior to the FCN algorithm and asymmetric 3D residual U-Net algorithm.

### 3.3. Evaluation Indicators of Segmentation Outcomes

**Figure 7:**
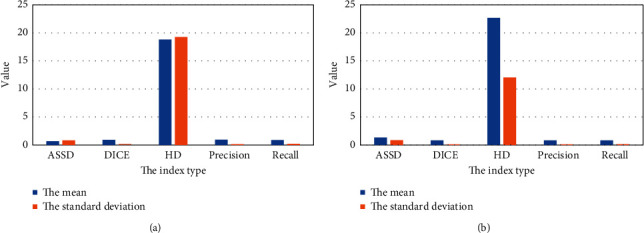
The evaluation indexes of segmentation results. (a) training set; (b) validation set.

As shown in Figure 7, for the training set, the mean values of ASSD, DICE, HD, accuracy, and sensitivity under the cascaded 3D deep residual network algorithm were 0.65, 0.912, 18.66, 0.93, and 0.90, respectively, and the standard deviations were 0.81, 0.04, 19.27, 0.07, and 0.07, respectively, while the corresponding mean values of the validation set are 1.31, 0.82, 22.59, 0.80, and 0.80, and the corresponding standard deviations were 0.85, 0.12, 12.07, 0.11, and 0.15, respectively. Evidently, the cascaded 3D deep residual network algorithm had high sensitivity and accuracy in segmentation of multimodal MRI images of stroke patients.

### 3.4. Comparison of Segmentation Results of Various Algorithms

**Figure 8:**
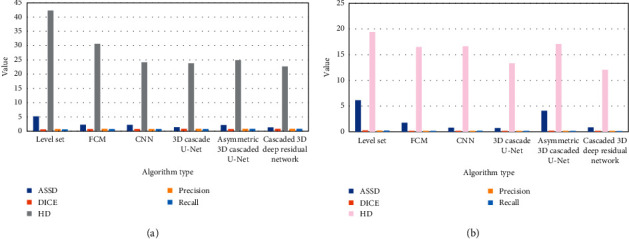
The segmentation outcomes of various algorithms on the test set. (a) mean value; (b) standard deviation.

**Figure 9:**
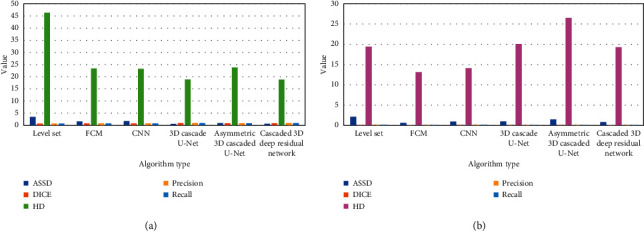
The segmentation outcomes of various algorithms on the validation set. (a) mean value; (b) standard deviation.

According to Figures 8 and 9, the cascaded 3D deep residual network algorithm had better results in terms of DICE, sensitivity, ASSD, and HD, both in the training set and the verification set. Taken together, the cascaded 3D deep residual network algorithm was superior to other algorithms in the segmentation of multimodal MRI images of stroke patients.

## 4. Discussion

Cardiovascular and cerebrovascular diseases have become the first cause of deaths in China. Studies have shown that the mortality rate of cardiovascular diseases in China is as high as 271/100,000. Among them, brain diseases are the most common and frequent. Brain diseases are usually divided into acute and chronic diseases. Acute brain diseases are characterized by a rapid onset, a high mortality rate, and a high disability rate [10], and stroke is a representative brain disease. The incidence of stroke is relatively high in China, and clinical statistics have revealed that as many as 3 million people die from strokes in China every year [11]. Stroke can be divided into two types: ischemic and hemorrhagic stroke, and the ischemic stroke is more common, accounting for 87% [12].

Stroke is a brain disease arising from long-term cerebral ischemia or hypoxia. Studies have shown that if the blood and oxygen supply to the brain can be restored in time, the brain damage can be minimized and the prognosis of the patients can be greatly improved. Therefore, early and accurate diagnosis of stroke is important. Multimodal MRI is currently a main method for doctors to diagnose stroke in clinic. Compared with other imaging technologies, it has many advantages such as comprehensive and accurate reflection of brain information, easy operation, and fast diagnosis. Therefore, it has been widely used in clinical practice. In this study, the stroke patients were diagnosed based on the multimodal MRI images. Studies have found that multimodal MRI can display the brain condition more comprehensively, accurately, and quickly.

As artificial intelligence and big data march forward continuously, they have gradually found applications in the medical field. How to extract effective features from massive medical images has become the focus of current research. Deep learning is an emerging medical image data processing method. Traditional learning algorithms have limited applications because of their weak feature expression ability due to the shallow structure of the training model [13], while deep learning makes up for the shortcomings of traditional learning algorithms because it is equipped with an advanced central processing unit, an improved machine algorithm, and massive medical images. It transforms the manual feature extraction into automatic machine extraction, reducing manual labor and heightening the accuracy of feature extraction. Based on the abovementioned advantages, it has received extensive attention [14]. According to whether the training data set is labeled or not, machine learning algorithms can be divided into two categories: supervised learning and unsupervised learning. Research has found that the supervised deep learning algorithm performs better in image analysis. In particular, the Conv.Net is currently the most valuable and potential method for image processing and analysis [15]. It mainly consists of the input layer, convolutional layer, downsampling layer, fully connected layer, and output layer. Gradually, a variety of deep CNN models have been established. Statistics have shown that their feature recognition error rate has been reduced to 3.5%. They are extensively used in image segmentation, image classification, image registration, and target detection [16, 17]. In the study, the cascaded 3D deep residual network algorithm was applied to segment multimodal MRI images of stroke patients, which demonstrated great capacities in foci segmentation of stroke patients.

## 5. Conclusions

In this study, the cascaded 3D deep residual network algorithm was applied to segment multimodal MRI images of patients with the ischemic stroke. It was found that the multimodal MRI images can provide comprehensive information and can assist doctors in diagnosis. The cascaded 3D deep residual network algorithm demonstrated great capabilities in foci segmentation. However, some limitations should be noted in the study. Due to the word limitation, the research is not in-depth enough. In the follow-up, an expanded sample size is necessary for further analyses.

## Data Availability

The data used to support the findings of this study are available from the corresponding author upon request.
